# Attitudes and perspectives of 534 Chinese pediatricians toward internet hospitals

**DOI:** 10.3389/fped.2022.948788

**Published:** 2022-09-28

**Authors:** Wenbin Cui, Weijun Zhu, Xiaojie Li, Danmai Wu, Ping He, Guangjun Yu

**Affiliations:** ^1^School of Public Health, Shanghai Jiao Tong University School of Medicine, Shanghai, China; ^2^Shanghai Children’s Hospital, Shanghai Jiao Tong University School of Medicine, Shanghai, China; ^3^Shanghai Hospital Development Center, Shanghai, China

**Keywords:** internet hospital, pediatrician, medical resource, perspective, China

## Abstract

**Background:**

Internet hospitals introduced in China have effectively reduced service time and space, promoted high-quality pediatric medical resources to grassroots areas, solved the contradiction between supply and demand of pediatric medical resources, and met patients’ increasing multi-level and diversified medical service needs. However, pediatricians’ attitudes toward and satisfaction with the use of internet hospitals remain unknown.

**Objective:**

This study aimed to investigate pediatricians’ knowledge of, use of, and satisfaction with internet hospitals in order to identify major issues in internet hospital development, and to understand pediatricians’ attitudes and opinions on the construction, development, and use of internet hospitals.

**Materials and methods:**

A total of 625 pediatricians in 17 public tertiary hospitals in Shanghai were surveyed from November 1–30, 2021. Five hundred and thirty four pediatricians completed the survey, and the response rate was 85.44%. Pediatricians’ baseline demographic data were collected and information about their use of and satisfaction with internet hospitals.

**Results:**

About 70.22% (375/534) of pediatricians knew about internet hospitals and about 54.68% (292/534) use internet hospitals for patient consultation, diagnosis, and treatment. Utilized services mainly focused on online consultation (271/292, 92.81%), online follow-up consultation (174/292, 59.59%), and health sciences (111/292, 38.01%). Online services were provided by 69.18% (202/292) of pediatricians for less than 1 h a day, and 75.00% (219/292) responded to fewer than five patient consultations online every day. Pediatricians’ overall satisfaction with internet hospitals was low (3.59 ± 0.92 points), user experience, systems functions, operation processes, service prices, and performance rewards of internet hospitals were main influencing factors. Pediatricians are enthusiastic about further development of internet hospitals, with 87.83% (469/534) willing to provide services on the internet hospital platform.

**Conclusion:**

Most pediatricians view internet hospitals favorably and are eager to contribute to the development of online diagnosis and treatment services. The development of internet hospitals will be more strongly supported by improving pediatricians’ satisfaction and mobilizing their enthusiasm and initiative to participate in internet medical services.

## Introduction

Short-staffing of pediatricians has always been a prominent problem in the medical community in China (1). The “White Paper of Pediatric Resources in China” shows a manpower shortage of more than 200,000 pediatricians at present (2), making the supply of pediatric medical services unbalanced and insufficient (3). Pediatric diagnosis and treatment resources are concentrated in children’s specialized hospitals and in the pediatrics departments of some comprehensive tertiary hospitals, showing that pediatrics is relatively weak at the grassroots level. Differences between regional, urban and rural areas are large (4). In recent years, China has actively promoted the development of internet medical care, promoted high-quality pediatric medical resources at the grassroots level, and helped to resolve the supply-demand gap for pediatric medical resources (5). Internet medical services have removed time and space constraints, allowing doctors and patients to consult at any time and from any location, while also improving resource distribution efficiency (6–8).

In the face of major epidemics, this efficient resource allocation method can compensate for a lack of offline medical resources in a timely and effective manner (9). Internet medical care, with its unique benefits, has greatly met the urgent needs of preventing the spread of epidemics (10). The method of diagnosis and treatment not only reduces the likelihood of cross-infection in patients with mild symptoms and realizes the “physical isolation” of patients’ behavior relative to medical treatment (11), but it also breaks down geographic barriers to medical resources and promotes the efficient allocation of medical resources (12). Aside from diagnosis, internet medical care fills the void left by family doctors in epidemic prevention and control, as well as providing timely consultation, triage, emotional comfort, psychological counseling, and other services to community residents (13).

The COVID-19 outbreak has served as a “catalyst” for China’s internet medical care industry. Since 2020, the number of internet hospitals in China has exploded, and public hospitals have begun to build their own internet hospitals to offer patients online consultation, online follow-up, electronic prescriptions, drug delivery, and other medical care services (14). The internet hospitals have been instrumental in meeting patients’ basic medical service needs (15), alleviating their anxiety about seeking medical treatment, reducing crowding and the risk of cross-infection (16), increasing the efficiency and safety of prevention and control work (17), and innovating the medical service model, thereby saving patients significant healthcare costs (18). Several studies have been conducted to date on the current state and characteristics of internet hospital construction in China (19, 20), or examined patients’ intention to use an internet hospital (21–23). However, few studies have examined pediatricians’ attitudes and opinions toward internet hospitals, nor have they examined the limiting factors hindering the growth of internet hospitals. Thus, in order to gain a true and objective understanding of pediatricians’ knowledge, use, and satisfaction with internet hospitals, we conducted this survey, aiming to identify the limiting factors impeding the development of internet hospitals. Better understanding of pediatricians’ attitudes and opinions on the construction, development and subsequent use of internet hospitals will help to make recommendations for mobilizing pediatricians’ enthusiasm and initiative, improving internet health services and promoting the development of internet hospitals, with an ultimate goal of resolving the issue of unequal access to pediatric medical care in China.

## Materials and methods

### Participants

A total of 625 pediatricians in 17 public tertiary hospitals in Shanghai were surveyed from November 1–30, 2021. All hospitals, including 14 general hospitals and three children’s specialty hospitals, had obtained internet hospital licenses. Using cluster sampling method, we surveyed pediatricians in pediatric departments of general hospitals, and the departments of respiratory, gastroenterology, cardiology, neurology, nephrology, endocrinology, dermatology, traditional Chinese medicine, children’s health care, and rehabilitation in children’s specialty hospitals. Inclusion criteria were: Pediatricians in practice. Exclusion criteria were: non-physician staff such as nursing, medical technology, and management, as well as non-hospital staff such as training students, advanced students, and interns. A total of 534 pediatricians completed the survey, and the questionnaire response rate was 85.44%.

### Data collection

A survey questionnaire was designed to collect data of pediatricians’ awareness, use, and satisfaction with internet hospitals, as well as their attitudes and views about internet hospitals. The questionnaire was divided into five sections, as follows:

• Demographic information: Gender, age, education level, job title, and length of service.
• Awareness: Pediatricians’ understanding of internet hospitals, including their types, service functions, value and management structure, and others.
• Utilization status: Pediatricians’ willingness to provide internet medical services, types of services they provide, length of time they serve each day, number of patients served each time, and the average time it takes to serve a patient.
• Satisfaction level: Pediatrician experience, system functions, operating procedures, working modes, working hours, patients’ charges, and performance rewards. Satisfaction survey was scored using a Likert five-point scale ranging from “extremely unsatisfied” to “extremely satisfied.” Satisfaction is quantified on a scale of 1 to 5.
• Attitudes and suggestions: Pediatricians’ attitudes toward internet hospitals, current difficulties, and future suggestions.

### Statistical analysis

Data were managed using Excel 2016 and all statistical analyses were done using SPSS 26.0 statistical software (IBM Corp. Armonk, NY, USA). Enumeration data are expressed as a rate or composition ratio, measurement data are shown as mean ± SD, and descriptive analysis, chi-square test, ANOVA, and multivariate analysis were used as statistical methods. α = 0.05, *P* < 0.05 indicated statistically significant differences.

## Results

### Participants’ baseline characteristics

Among 534 pediatricians surveyed, as shown in Table 1, 169 were general hospital pediatricians and 365 were children’s specialty hospital pediatricians, 292 pediatricians had participated in online services. Female pediatricians were significantly more prevalent in both general hospitals (125/169, 73.96%) and children’s specialty hospitals (263/365, 72.05%). And the proportion of male pediatricians who participated in online services was higher than those who did not (*P* = 0.006).

**TABLE 1:** Demographic characteristics of the participants (*N* = 534).

Characteristics	Total value	Different types of hospitals			Participated in online services		
		General hospital	Specialized hospital	*P*-value	Yes	No	*P*-value
Sample size, *n*	534	169	365		292	242	
**Gender, *n* (%)**				0.645			0.006
Male	146 (27.34)	44 (26.04)	102 (27.95)		94 (32.19)	52 (21.49)	
Female	388 (72.66)	125 (73.96)	263 (72.05)		198 (67.81)	190 (78.51)	
**Age, *n* (%)**				<0.001			0.002
≤25	23 (4.31)	9 (5.33)	14 (3.84)		8 (2.74)	15 (6.20)	
26–35	239 (44.76)	73 (43.20)	166 (45.48)		116 (39.73)	123 (50.83)	
36–45	182 (34.08)	42 (24.85)	140 (38.36)		112 (38.36)	70 (28.93)	
46–55	69 (12.92)	33 (19.53)	36 (9.86)		47 (16.10)	22 (9.09)	
>55	21 (3.93)	12 (7.10)	9 (2.47)		9 (3.08)	12 (4.96)	
**Education level, *n* (%)**				0.033			
College education	29 (5.43)	16 (9.47)	13 (3.56)		11 (3.77)	18 (7.44)	0.035
Bachelor degree	95 (17.79)	32 (18.93)	63 (17.26)		43 (14.73)	52 (21.49)	
Master’s degree	259 (48.50)	74 (43,79)	185 (50.68)		149 (51.03)	110 (45.45)	
Doctoral degree	151 (28.28)	47 (27.81)	104 (28.49)		89 (30.48)	62 (25.62)	
**Title, *n* (%)**				0.038			<0.001
Primary title	184 (34.46)	63 (37.28)	121 (33.15)		69 (23.63)	115 (47.52)	
Middle title	225 (42.13)	60 (35.50)	165 (45.21)		141 (48.29)	84 (34.71)	
Vice-senior title	81 (15.17)	25 (14.79)	56 (15.34)		54 (18.49)	27 (11.16)	
Senior title	44 (8.24)	21 (12.43)	23 (6.30)		28 (9.59)	16 (6.61)	
**Work experience, *n* (%)**				0.128			0.003
Less than 3 years	67 (12.55)	24 (14.20)	43 (11.78)		23 (7.88)	44 (18.18)	
3–8 years	163 (30.52)	44 (26.04)	119 (32.60)		88 (30.14)	75 (30.99)	
8–15 years	139 (26.03)	39 (23.08)	100 (27.40)		84 (28.77)	55 (22.73)	
More than 15 years	165 (30.90)	62 (36.69)	103 (28.22)		97 (33.22)	68 (28.10)	

The proportion of pediatricians aged over 45 years in general hospitals (45/169, 26.63%) was significantly higher than that in children’s specialized hospitals (45/365, 12.33%) (*P* < 0.001). And the proportion of those aged over 35 years who participated in online services (168/292, 57.53%) was higher than those who did not (104/365, 42.96%) (*P* = 0.002).

The majority of the 534 pediatricians have a master’s degree or higher, and the proportion of those with advanced degrees was higher in children’s specialized hospitals (289/365, 79.18%) than that in general hospitals (121/169, 71.60%) (*P* = 0.033), the same is true among who participate in online service (238/292, 81.51%) and who did not (172/242, 71.07%) (*P* = 0.035).

The proportion of pediatricians with senior professional titles is also high, and senior pediatricians in general hospitals (21/169, 12.43%) outnumbered those in children’s specialty hospitals (23/365, 6.30%) (*P* = 0.038). And the proportion of those with middle title or higher who participated in online services (223/293, 76.37%) was significantly higher than those who did not (127/242, 52.48%) (*P* < 0.001). And the proportion of pediatricians with over 8 years of working experience who participated in online services (181/292, 64.18%) was higher than those who did not (123/242, 50.83%) (*P* = 0.003).

### Awareness of internet hospitals among study participants

Results in Table 2 indicated that the majority of 534 pediatricians were very familiar with and understand internet hospitals (375/534, 70.22%), and pediatricians in children’s specialized hospitals and who participated in online services have a better understanding of internet hospitals than pediatricians in general hospitals (*P* = 0.005) and who did not provide online services (*P* < 0.001).

**TABLE 2:** Awareness of internet hospitals among study participants (*N* = 534).

Characteristics	Total value	Different types of hospitals			Participated in online services		
		General hospital	Specialized hospital	*P*-value	Yes	No	*P*-value
Sample size, *n*	534	169	365		292	242	
**Degree of knowledge of internet hospitals, *n* (%)**				0.005			<0.001
Very familiar with	138 (25.84)	39 (23.08)	99 (27.12)		101 (34.59)	37 (15.29)	
Know a better bit	237 (44.38)	64 (37.87)	173 (47.40)		134 (45.89)	103 (45.56)	
Only heard of	142 (26.59)	56 (33.14)	86 (23.56)		55 (18.84)	87 (35.95)	
Never heard of	17 (3.18)	10 (5.92)	7 (1.92)		2 (0.68)	15 (6.20)	
**Ways of learning about internet hospitals, *n* (%)**				0.029			0.369
Internal training	451 (84.46)	129 (76.33)	322 (88.22)		263 (90.07)	188 (77.69)	
Media reports	266 (49.81)	97 (57.40)	169 (46.30)		140 (47.95)	126 (52.07)	
Advertising	174 (32.58)	71 (42.01)	103 (28.22)		99 (33.90)	75 (30.99)	
Friends and colleagues	225 (42.13)	81 (47.93)	144 (39.45)		117 (40.07)	108 (44.63)	
Conference lectures	149 (27.90)	48 (28.40)	101 (27.67)		88 (30.14)	61 (25.21)	
**Types of internet hospitals, *n* (%)**				0.109			0.481
Hospital-led	444 (83.15)	121 (71.60)	323 (88.49)		251 (85.96)	193 (79.75)	
Cooperation construction	343 (64.23)	115 (68.05)	228 (62.47)		207 (70.89)	136 (56.20)	
Enterprise-platform	477 (89.33)	155 (91.72)	322 (88.22)		270 (92.47)	207 (85.54)	
**Hospital information systems are interconnected, *n* (%)**				0.567			<0.001
Yes	238 (44.57)	73 (43.20)	165 (45.21)		134 (45.89)	104 (42.98)	
No	49 (9.18)	13 (7.69)	36 (9.86)		43 (14.73)	6 (2.48)	
Not clear	247 (46.25)	83 (49.11)	164 (44.93)		115 (39.38)	132 (54.55)	
**Independent management department is set up, *n* (%)**				0.489			0.007
Yes	236 (44.19)	69 (40.83)	167 (45.75)		146 (50.00)	90 (37.19)	
No	18 (3.37)	5 (2.96)	13 (3.56)		11 (3.77)	7 (2.89)	
Not clear	280 (52.43)	95 (56.21)	185 (50.68)		135 (46.23)	145 (59.92)	
**Role of internet hospitals, *n* (%)**				0.905			0.976
Overcome geographic constraints	487 (91.20)	155 (91.72)	332 (90.96)		272 (93.15)	215 (88.84)	
Save medical costs	469 (87.83)	146 (86.39)	323 (88.49)		260 (89.04)	209 (86.36)	
Expand medical services	430 (80.52)	146 (86.39)	284 (77.81)		246 (84.25)	184 (76.03)	
Improve resource allocation	434 (81.27)	143 (84.62)	291 (79.73)		239 (81.85)	195 (80.58)	
Improve accessibility of healthcare	383 (71.72)	127 (75.15)	256 (70.14)		215 (73.63)	168 (69.42)	

Internal hospital training (451/534, 84.46%), media reports (266/534, 49.81%), friends or colleagues (225/534, 42.13%), advertising (174/534, 32.58%), conference lectures (149/534, 27.90%), and other channels were the most common ways pediatricians learned about internet hospitals. The proportion of pediatricians in general hospitals who learned about internet hospitals from outside sources such as advertisements, media reports, friends or colleagues was significantly higher than that in children’s specialist hospitals (*P* = 0.029).

The majority of the 534 pediatricians surveyed (444, 83.15%) were aware of public hospital-led internet hospitals and enterprise platform internet hospitals (477, 89.33%). Pediatricians frequently work in specialized hospitals for children. The awareness ratio of these two types of internet hospitals was very close, and the awareness ratio of general hospital pediatricians to enterprise-platform internet hospitals (155/169, 91.72%) was significantly higher than that of public hospital-led internet hospitals (121/169, 71.60%).

When asked whether the internet hospital and the various business information systems within the hospital were connected and whether a separate department was established to oversee the operation and management of the internet hospital, more than half of pediatricians expressed uncertainty or provided a negative response, especially those who had not yet participated in online services.

Regarding the role of internet hospitals, 91.20% (487/534) of pediatricians believed that the hospitals could overcome the time and geographic constraints of conventional medical services, while 87.83% (469/534) believed that the hospitals could save patients time and transportation costs; 81.27% (434/534) of pediatricians believed that the allocation and use of medical resources could be improved; and 80.52% (430/534) of pediatricians believed that the hospitals could expand medical services to improve patient safety.

### Current usage of internet hospitals among study participants

Survey results (see Table 3) revealed that 54.68% (292/534) of pediatricians have used internet hospitals to provide patient care, and the proportion of pediatricians working in children’s specialized hospitals (210/365, 57.53%) was higher than the proportion working in general hospitals (82/169, 48.52%). Pediatricians’ use of internet hospitals is associated with gender, age, education, professional title, and length of service, and statistically significant differences are shown between these factors. The proportion of male pediatricians (94/146, 64.38%) was higher than that of female pediatricians (198/388, 51.03%) (*P* = 0.006). The proportion of pediatricians aged 35–45 and 45–55 was higher than the other age groups (*P* = 0.002), as was the proportion of pediatricians with master’s and doctoral degrees (*P* = 0.035), intermediate titles and above (*P* < 0.001), and 8–15 years of service (*P* = 0.003) compared to other years of service.

**TABLE 3:** Current usage of internet hospitals by study participants (*N* = 534).

Characteristics	Total value	Launched online services		*P*-value
		Yes	No	
Sample size, *n*	534	292	242	
**Category, *n* (%)**				0.052
General hospital	169 (31.65)	82 (48.52)	87 (51.48)	
Specialized hospital	365 (68.35)	210 (57.53)	155 (42.47)	
**Gender, *n* (%)**				0.006
Male	146 (27.34)	94 (64.38)	52 (35.62)	
Female	388 (72.66)	198 (51.03)	190 (48.97)	
**Age, *n* (%)**				0.002
≤25	23 (4.31)	8 (34.78)	15 (65.22)	
26–35	239 (44.76)	116 (48.54)	123 (51.46)	
36–45	182 (34.08)	112 (61.54)	70 (38.46)	
46–55	69 (12.92)	47 (68.12)	22 (31.88)	
>55	21 (3.93)	9 (42.86)	12 (57.14)	
**Education level, *n* (%)**				0.035
College education	29 (5.43)	11 (37.93)	18 (62.07)	
Bachelor degree	95 (17.79)	43 (45.26)	52 (54.74)	
Master’s degree	259 (48.50)	149 (57.53)	110 (42.47)	
Doctoral degree	151 (28.28)	89 (58.94)	62 (41.06)	
**Title, *n* (%)**				<0.001
Primary title	184 (34.46)	69 (37.50)	115 (67.50)	
Middle title	225 (42.13)	141 (62.67)	84 (37.33)	
Vice-senior title	81 (15.17)	54 (66.67)	27 (33.33)	
Senior title	44 (8.24)	28 (63.64)	16 (36.36)	
**Work experience, *n* (%)**				0.003
Less than 3 years	67 (12.55)	23 (34.33)	44 (65.67)	
3–8 years	163 (30.52)	88 (53.99)	75 (46.01)	
8–15 years	139 (26.03)	84 (60.43)	55 (39.57)	
More than 15 years	165 (30.90)	97 (58.79)	68 (41.21)	

### Online service content and duration

**FIGURE 1:**
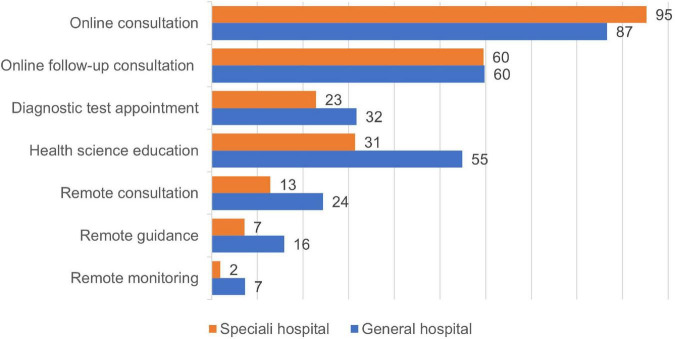
Internet hospital services provided by 292 pediatricians (%).

The vast majority of pediatricians (271/292, 92.81%) provided online consultations through internet hospitals, followed by online follow-up consultations (174/292, 59.59%), health science (111/292, 38.01%), examinations and laboratory appointments (74/292, 25.34%), teleconsultation (47/292, 16.10%), tele-guidance (28/292, 9.59%), and tele-monitoring (10/292, 3.42%). Furthermore, differences in terms of service items appeared to exist among pediatricians (Figure 1). The proportion of pediatricians in children’s specialized hospitals perform online consultations (200/210, 95.24%) was higher than that in general hospitals (71/82, 86.59%) (*P* = 0.010). Pediatricians at general hospitals perform health science education (45/82, 54.88%, *P* < 0.001), remote consultation (20/82, 24.39%, *P* = 0.016), remote guidance (13/82, 15.85%, *P* = 0,023), and remote monitoring (6/82, 7.32%, *P* = 0.032) services more often than the staff of children’s specialist hospitals.

**FIGURE 2:**
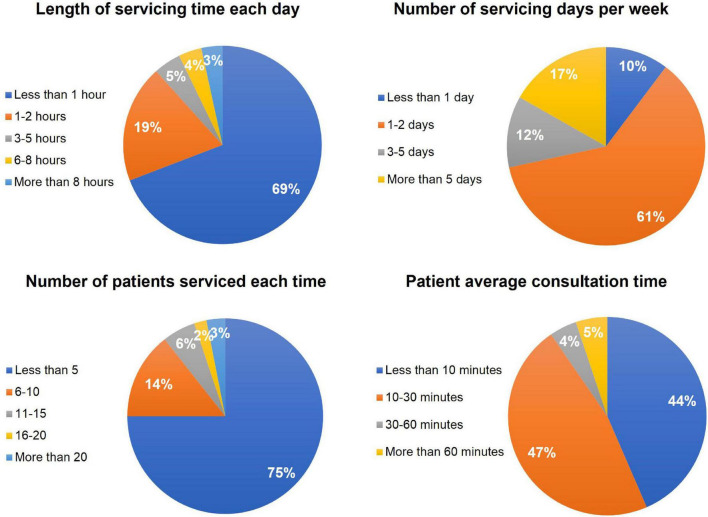
Online working time and number of patients served (*N* = 292).

In terms of service time, 69.18% (202/292) of pediatricians provide online medical services for less than an hour per day on average, and 61.30% (179/292) provide online medical services on an average of 1–2 days per week. In terms of volume, 75.00% (219/292) of pediatricians provide less than five online services per day on average, and 90.41% (264/292) of pediatricians provide service times of less than 30 min per person on average (Figure 2).

### Pediatricians’ satisfaction with internet hospitals

A survey of 292 pediatricians who had launched online services was conducted using the Likert scale, and respondents rated their satisfaction with internet hospitals as “very dissatisfied,” “dissatisfied,” “average,” “satisfied,” or “very satisfied.” The five responses were assigned a score of 1, 2, 3, 4, or 5. The mean score for 292 pediatricians was 3.59 (standard deviation 0.92), indicating that not everyone is pleased with the internet hospital. As illustrated in Table 4, the age and professional title of pediatricians were both significant influencing factors. Pediatricians younger than age 35 (*P* = 0.014) with junior professional titles (*P* = 0.007) are significantly more satisfied than those in the other groups.

**TABLE 4:** Pediatricians’ satisfaction with internet hospitals (*N* = 292).

Characteristics	*N* (%)	Mean ± SD	*F* value	*P*-value
Sample size	292 (100)	3.59 ± 0.92		
**Category**			0.000	0.994
General hospital	82 (48.52)	3.59 ± 0.83		
Specialized hospital	210 (57.53)	3.59 ± 0.95		
**Gender**			0.121	0.728
Male	94 (64.38)	3.56 ± 0.90		
Female	198 (51.03)	3.60 ± 0.93		
**Age**			3.204	0.014
≤25	8 (34.78)	3.59 ± 1.53		
26–35	116 (48.54)	3.81 ± 0.92		
36–45	112 (61.54)	3.39 ± 0.86		
46–55	47 (68.12)	3.54 ± 0.85		
>55	9 (42.86)	3.49 ± 0.86		
**Education level**			0.630	0.596
College education	11 (37.93)	3.69 ± 1.36		
Bachelor degree	43 (45.26)	3.73 ± 0.93		
Master’s degree	149 (57.53)	3.59 ± 0.90		
Doctoral degree	89 (58.94)	3.50 ± 0.89		
**Title**			4.166	0.007
Primary title	69 (37.50)	3.92 ± 0.90		
Middle title	141 (62.67)	3.48 ± 0.95		
Vice-senior title	54 (66.67)	3.47 ± 0.79		
Senior title	28 (63.64)	3.56 ± 0.89		
**Work experience**			1.779	0.151
Less than 3 years	23 (34.33)	3.61 ± 1.17		
3–8 years	88 (53.99)	3.77 ± 0.91		
8–15 years	84 (60.43)	3.50 ± 0.83		
More than 15 years	97 (58.79)	3.50 ± 0.92		

As illustrated in Table 5, the results of multi-factor analysis of variance showed that, user experience (*P* < 0.001), system functions (*P* < 0.001), operation processes (*P* < 0.001), duration of single consultation (*P* < 0.001), service prices (*P* < 0.001) and performance incentive (*P* < 0.001) of internet hospitals were factors that affected pediatricians’ satisfaction with internet hospitals, and that would impede the development of internet hospitals.

**TABLE 5:** Multivariate analysis of factors influencing pediatricians’ satisfaction with internet hospitals.

Variables	Sum of squares	Degrees of freedom	Mean squares	*F* ratio	*P*-value
User experience	0.658	3	0.219	182.528	<0.001
System functions	0.503	3	0.168	139.454	<0.001
Operation processes	0.812	3	0.271	225.149	<0.001
Duration of single consultation	1.317	4	0.329	273.824	<0.001
Service prices	0.653	4	0.163	135.703	<0.001
Performance incentive	0.781	4	0.195	162.389	<0.001

### Problems of internet hospitals

**FIGURE 3:**
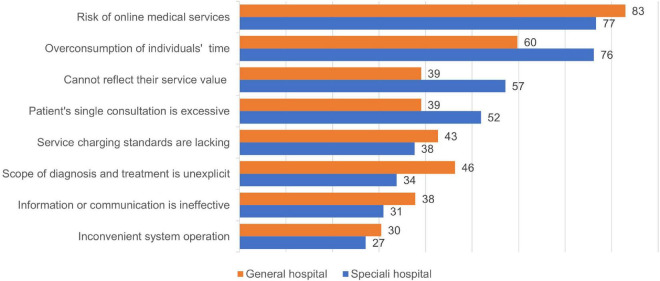
Problems of internet hospitals (%).

The 292 pediatricians surveyed who had launched internet medical services generally believed that internet hospitals have three main problems, including: risk of online medical services (229/292, 78.42%); overconsumption of individuals’ working and living time (209/292, 71.58%); and not able to accurately reflect the value of their own services (52.05%, 152/292). Several other problems exist as well, including that the duration of the patient’s single consultation is excessive (141/292, 48.29%); service charging standards are lacking (114/292, 39.04%); the scope of diagnosis and treatment is not explicit (109/292, 37.33%); information or communication about receiving services is ineffective (96/292, 32.88%); and inconvenient system operation (82/292, 28.08%). As presented in Figure 3, pediatricians at children’s specialist hospitals believe that internet hospitals consume personal work and life time (160/210, 76.19%, *P* = 0.005) and cannot accurately reflect the value of their services (120/210, 57.14%, *P* = 0.005), and the proportion of patients with an excessively lengthy single consultation (109/210, 51.90%, *P* = 0.048) is higher than that of pediatricians at general hospitals.

### Pediatricians’ attitudes toward internet hospitals

**FIGURE 4:**
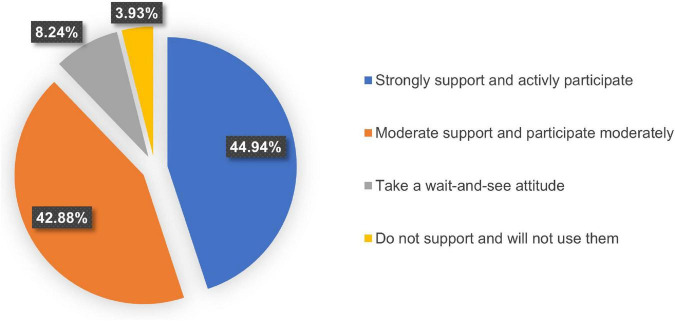
Pediatricians’ attitudes toward internet hospitals (%).

Among the 534 pediatricians surveyed, 44.94% (240/534) expressed strong support for internet hospitals and indicated that they would actively participate in various online services, while 42.88% (229/534) expressed moderate support for internet hospitals and indicated that they would also participate moderately. Among these, 8.24% (44/534) had a wait-and-see attitude toward the development of internet hospitals, while only 3.93% (21/534) were pessimistic about the development of internet hospitals and will not use them (Figure 4).

## Discussion

Findings of the present study show that the majority of pediatricians are women; the average age of pediatricians in general hospitals is older, the working years are longer and the proportion of doctors with senior titles is higher than in children’s specialized hospitals. The shortage of doctors is a common occurrence. In China, only 0.63 pediatricians are available for every 1,000 children. According to the “Implementation Plan of China National Program for Women’s and Child Development (2021–2030),” the number of practicing (assistant) pediatricians per thousand children is expected to reach 1.12 by 2030 (24), but a significant gap still exists compared with developed countries (25, 26). This gap can be explained by the high workload, high occupational risks, and low pay of these assistant practicing pediatricians (1). These factors make students entering medical school hesitant to pursue a career in pediatrics. As a result, the number of pediatrics students graduating from medical schools each year is limited, and the majority prefers to work in tertiary children’s hospitals, making it difficult for general hospitals to recruit new talent.

The Chinese government has issued a number of policy documents in recent years to encourage the development of internet medical care (14, 27). Simultaneously, with the development of modern information technologies such as the Internet of Things (IoT) big data, cloud computing, and 5G mobile networks, AI technology has been more widely adopted in the medical field (28, 29), internet hospitals have emerged as the times have demanded. Since 2020, the COVID-19 epidemic has accelerated the development of internet hospitals (11, 30), with various Chinese provinces actively building internet hospitals to provide telemedicine, health consultation, medical guidance, health education, psychological counseling, and other services (31). The increase in internet hospitals has played an important role in assisting epidemic research and judgment, innovating diagnosis and treatment models, and improving service efficiency (32) by leveraging the advantages of non-contact, rapid response, and breaking down geographical barriers (33). According to the findings of the present study, more than half of pediatricians have provided online services to patients *via* internet hospitals in the form of text, images, voice, and video. They generally believe that internet hospitals have broadened the content and scope of conventional medical services, improved the efficiency with which high-quality pediatric medical resources are allocated, effectively solved the contradiction between supply and demand for pediatric medical resources, and reduced the financial burden on patients’ families (34).

The present study discovered that China has two types of internet hospitals: public hospital-led internet hospitals and an enterprise-led internet hospital platform (35). Despite differences in essential characteristics, functional orientation, operation mode, and development prospects, both of these types provide patients with services such as remote diagnosis and treatment, rehabilitation guidance, chronic disease management, follow-up, and health knowledge popularization (13). We also found that public hospital pediatricians provide a variety of medical and health services to patients through various types of internet hospitals, which improves the fairness and accessibility of high-quality pediatric medical resources and creates a new service model that integrates online and offline services (18). A new system of patient-centered diagnosis and treatment services has been built, greatly improving medical service efficiency, broadening the scope of hospital business, and improving patient satisfaction and medical experience (36).

According to the present findings, the title and years of working experience of pediatricians are important influencing factors based on whether they provide internet medical services. The majority of pediatricians who provide online services have intermediate or higher titles and have been in practice for at least 3 years. The online service of internet hospitals has high requirements for the quality of pediatricians’ ability and their work experience, emphasizing the importance of improving pediatricians’ diagnosis and treatment capabilities, to ensure the quality of internet medical services and patient safety (37).

During the survey, we found that different pediatric specialties have different needs for internet hospital services. In addition to the online consultation, online follow-up, telemedicine and other services currently provided, some more online services are required, for example, the respiratory departments need remote home atomization, nephrology departments need remote monitoring and home hemodialysis, dermatology departments hope initial evaluation, diagnosis and treatment of the patient with simple skin diseases should be performed online, and so on. It gives us a hint: internet hospitals should enrich and innovate service items and functions to meet the needs of different pediatric specialists, and provide children with more convenient, diversified and personalized medical services.

Our results demonstrated that pediatricians have a limited amount of time to provide online services *via* internet hospitals. Most pediatricians work for internet hospitals no more than 2 days per week and no more than 2 h per day. The number of people who respond to patient consultations online is also fewer than ten. The average consultation time per patient is somewhat lengthy. At the moment, the development of internet hospitals faces numerous problems and challenges (7). We discovered that user experience, system functions, operational procedures, service prices, and performance rewards of internet hospitals are main factors influencing pediatricians’ overall satisfaction with internet hospitals. We recommend that the public hospitals should strengthen the construction of service platforms and optimize system operation processes to continuously improve user experience, and establish a long-term incentive mechanism to encourage pediatricians to actively participate in internet hospital services and improve pediatricians’ awareness of and satisfaction with internet hospitals, so as to make the online health services more available and accelerate the development of internet hospitals.

Furthermore, we found that pediatricians who have not yet launched online services for the following reasons: (1) Pediatricians are extremely busy with their daily duties, leaving little time or energy for online services; (2) Internet medical services lack pricing and charging standards, making it impossible to accurately reflect the value of pediatricians’ technical services; and (3) Online diagnosis and treatment services are limited to consultations for common and chronic diseases, with many other medical services unavailable online. Therefore, we suggest that the government should improve the price and charging standards of online service items, which should be combined with physicians’ professional title, service time, service forms, service scenarios, service intensity, etc. While also expand the content and scope of online services and clearly delineate the identities of patients with common and chronic diseases as soon as possible, so as to mobilize the enthusiasm and initiative of pediatricians to carry out internet medical services, and provide patients with all-round, full-process medical and health management services.

Moreover, internet hospitals are expected to grow rapidly as information technology is widely applied in the field of health care and relevant policies and regulations are constantly improved. However, at the moment, China’s internet hospitals are still in the early stages of development, and have not yet fully realized their advantageous role and value. Pediatricians are in a position to introduce internet hospitals to patients and to recommend the related internet services. In order to truly maximize the benefits of internet medical services, patients must be considered an important audience and, in addition to promoting the internet hospitals to physicians, we should also continue to improve patient use and satisfaction with internet hospitals (38).

### Limitations and future research

This study has several limitations. First, we only surveyed pediatricians at the top three tertiary public hospitals in Shanghai that have already opened internet hospitals, leaving out district-level public hospitals, grass-roots community health service centers, and enterprise-platform internet hospitals. Second, the present survey was conducted in Shanghai, where the allocation of pediatric medical resources is relatively abundant, and we did not investigate other regions in China, particularly remote areas with a scarcity of high-quality pediatric medical resources. Third, we only polled internet medical service providers (physicians) rather than internet medical service recipients (patients). As a result, the representativeness of the survey results is limited, and some biases may exist. It is possible that other functions, effects, or issues with internet hospitals are not being addressed. Future research should broaden the survey’s scope and examine various types of public medical institutions and platform-based internet hospitals in different areas of China. In addition to medical personnel, surveys of patients are required to gain an overall understanding of cognitive use and satisfaction with internet hospitals, as well as patients’ attitudes and views on promoting the construction and development of internet hospitals.

## Conclusion

In conclusion, most pediatricians view internet hospitals favorably and are eager to contribute to the development of online diagnosis and treatment services. Pediatricians play an important role in providing pediatric internet diagnosis and treatment services and are frequently able to influence patients’ choices of medical service content, methods, and procedures. The development of internet hospitals will be greatly aided by effectively improving pediatricians’ satisfaction and mobilizing their enthusiasm and initiative to participate in internet medical services.

## Data availability statement

The original contributions presented in this study are included in the article/supplementary material, further inquiries can be directed to the corresponding authors.

## Author contributions

WC, PH, and GY conceived and designed the study. WC, WZ, DW, and XL contributed to the literature research, data collection, data analysis, result interpretation, and manuscript preparation. PH and GY supervised, reviewed, and revised the manuscript. All authors contributed to the article and approved the submitted version.

## References

[1] Zhang Y Huang L Zhou X Zhang X Ke Z Wang Z Characteristics and workload of pediatricians in China. *Pediatrics.* (2019) 144:e20183532. 10.1542/peds.2018-3532 31253739

[2] Wu Q Zhao L Ye XC. Shortage of healthcare professionals in China. *BMJ.* (2016) 354:i4860. 10.1136/bmj.i4860 27659864

[3] Shi J Yan X Wang M Lei P Yu G. Factors influencing the acceptance of pediatric telemedicine services in China: a cross-sectional study. *Front Pediatr.* (2021) 9:745687. 10.3389/fped.2021.745687 34733810 PMC8558490

[4] Zhang X Wang J Huang LS Zhou X Little J Hesketh T Associations between measures of pediatric human resources and the under-five mortality rate: a nationwide study in China in 2014. *World J Pediatr.* (2021) 17:317–25. 10.1007/s12519-021-00433-0 34097241 PMC8183000

[5] Wang H Liang L Du C Wu Y. Implementation of online hospitals and factors influencing the adoption of mobile medical services in China: cross-sectional survey study. *JMIR MHealth UHealth.* (2021) 9:e25960. 10.2196/25960 33444155 PMC7869921

[6] Qiu Y Liu Y Ren W Qiu Y Ren J. Internet-based and mobile-based general practice: cross-sectional survey. *J Med Internet Res.* (2018) 20:e266. 10.2196/jmir.8378 30257819 PMC6300040

[7] He C Zhou Q Chen W Tian J Zhou L Peng H Using an internet-based hospital to address maldistribution of health care resources in rural areas of guangdong province, China: retrospective and descriptive study. *JMIR Med Inform.* (2018) 6:e51. 10.2196/medinform.9495 30578195 PMC6320436

[8] Lv Q Jiang Y Qi J Zhang Y Zhang X Fang L Using mobile apps for health management: a new health care mode in China. *JMIR MHealth UHealth.* (2019) 7:e10299. 10.2196/10299 31162131 PMC6682298

[9] Ye Q Deng Z Chen Y Liao J Li G Lu Y. How resource scarcity and accessibility affect patients’ usage of mobile health in China: resource competition perspective. *JMIR MHealth UHealth.* (2019) 7:e13491. 10.2196/13491 31400104 PMC6707027

[10] Gong K Xu Z Cai Z Chen Y Wang Z. Internet hospitals help prevent and control the epidemic of COVID-19 in China: multicenter user profiling study. *J Med Internet Res.* (2020) 22:e18908. 10.2196/18908 32250962 PMC7159055

[11] Hong Z Li N Li D Li J Li B Xiong W Telemedicine during the COVID-19 pandemic: experiences from Western China. *J Med Internet Res.* (2020) 22:e19577. 10.2196/19577 32349962 PMC7212818

[12] Deng Z Hong Z Ren C Zhang W Xiang F. What predicts patients’ adoption intention toward mHealth services in China: empirical study. *JMIR MHealth UHealth.* (2018) 6:e172. 10.2196/mhealth.9316 30158101 PMC6135967

[13] Han Y Lie RK Guo R. The internet hospital as a telehealth model in China: systematic search and content analysis. *J Med Internet Res.* (2020) 22:e17995. 10.2196/17995 32723721 PMC7424477

[14] Xu X Cai Y Wu S Guo J Yang L Lan J Assessment of internet hospitals in China during the covid-19 pandemic: national cross-sectional data analysis study. *J Med Internet Res.* (2021) 23:e21825. 10.2196/21825 33417586 PMC7819672

[15] Sun S Yu K Xie Z Pan X. China empowers internet hospital to fight against COVID-19. *J Infect.* (2020) 81:e67–8. 10.1016/j.jinf.2020.03.061 32251688 PMC7129532

[16] Xu X Wu X Jiang X Xu K Ying L Ma C Clinical findings in a group of patients infected with the 2019 novel coronavirus (SARS-Cov-2) outside of Wuhan, China: retrospective case series. *BMJ.* (2020) 368:m606. 10.1136/bmj.m606 32075786 PMC7224340

[17] He D Gu Y Shi Y Wang M Lou Z Jin C. COVID-19 in China: the role and activities of internet-based healthcare platforms. *Glob Health Med.* (2020) 2:89–95. 10.35772/ghm.2020.01017 33330783 PMC7731100

[18] Tu J Wang C Wu S. The internet hospital: an emerging innovation in China. *Lancet Glob Health.* (2015) 3:e445–6. 10.1016/S2214-109X(15)00042-X 26187488 PMC7129805

[19] Xie X Zhou W Lin L Fan S Lin F Wang L Internet hospitals in china: cross-sectional survey. *J Med Internet Res.* (2017) 19:e239. 10.2196/jmir.7854 28676472 PMC5516104

[20] Lai Y Chen S Li M Ung COL Hu H. Policy interventions, development trends, and service innovations of internet hospitals in china: documentary analysis and qualitative interview study. *J Med Internet Res.* (2021) 23:e22330. 10.2196/22330 34283025 PMC8335616

[21] Li D Hu Y Pfaff H Wang L Deng L Lu C Determinants of patients’ intention to use the online inquiry services provided by internet hospitals: empirical evidence from China. *J Med Internet Res.* (2020) 22:e22716. 10.2196/22716 33006941 PMC7599063

[22] Li P Luo Y Yu X Wen J Mason E Li W Patients’ perceptions of barriers and facilitators to the adoption of e-hospitals: cross-sectional study in western China. *J Med Internet Res.* (2020) 22:e17221. 10.2196/17221 32525483 PMC7317627

[23] Zhou M Zhao L Kong N Campy KS Qu S Wang S. Factors influencing behavior intentions to telehealth by Chinese elderly: an extended TAM model. *Int J Med Inform.* (2019) 126:118–27. 10.1016/j.ijmedinf.2019.04.001 31029253

[24] National Health Commission of the People’s Republic of China. *Implementation Plan of China National Program for Women’s and Child Development (2021-2030).* (2022). Available online at: http://www.gov.cn/zhengce/zhengceku/2022-04/09/content_5684258.htm (accessed Apr. 02, 2022).

[25] Sakai R Fink G Kawachi I. Pediatricians’ practice location choice-Evaluating the effect of Japan’s 2004 postgraduate training program on the spatial distribution of pediatricians. *J Epidemiol.* (2014) 24:239–49. 10.2188/jea.je20130117 24681844 PMC4000772

[26] Michelson KA Cushing AM Bucholz EM. Association of county-level availability of pediatricians with emergency department visits. *Pediatr Emerg Care.* (2022) 38:e953–7. 10.1097/PEC.0000000000002502 34282091 PMC8770659

[27] Shi A Zhou X Xie Z Mou H Ouyang Q Wang D. Internet plus health care’s role in reducing the inequality of high-quality medical resources in China. *Asia Pac J Public Health.* (2021) 33:997–8. 10.1177/10105395211044954 34514860

[28] Lu ZX Qian P Bi D Ye ZW He X Zhao YH Application of AI and IoT in clinical medicine: summary and challenges. *Curr Med Sci.* (2021) 41:1134–50. 10.1007/s11596-021-2486-z 34939144 PMC8693843

[29] Mbunge E Akinnuwesi B Fashoto SG Metfula AS Mashwama P. A critical review of emerging technologies for tackling COVID-19 pandemic. *Hum Behav Emerg Technol.* (2020) 1:10.1002/hbe2.237. 10.1002/hbe2.237 33363278 PMC7753602

[30] Wu J Xie X Yang L Xu X Cai Y Wang T Mobile health technology combats COVID-19 in China. *J Infect.* (2021) 82:159–98. 10.1016/j.jinf.2020.07.024 32730998 PMC7384407

[31] Zhi L Yin P Ren J Wei G Zhou J Wu J Running an internet hospital in china: perspective based on a case study. *J Med Internet Res.* (2021) 23:e18307. 10.2196/18307 34342267 PMC8485192

[32] Yang F Shu H Zhang X. Understanding “Internet Plus Healthcare” in China: policy text analysis. *J Med Internet Res.* (2021) 23:e23779. 10.2196/23779 34309581 PMC8367124

[33] Wu H Lu N. Online written consultation, telephone consultation and offline appointment: An examination of the channel effect in online health communities. *Int J Med Inform.* (2017) 107:107–19. 10.1016/j.ijmedinf.2017.08.009 29029686

[34] Marcin JP Shaikh U Steinhorn RH. Addressing health disparities in rural communities using telehealth. *Pediatr Res.* (2015) 79:169–76. 10.1038/pr.2015.192 26466080

[35] Liu L Shi L. Chinese patients’ intention to use different types of internet hospitals: cross-sectional study on virtual visits. *J Med Internet Res.* (2021) 23:e25978. 10.2196/25978 34397388 PMC8398707

[36] Zhai Y Ge X Liu X Xie L Shen Q Ye C An internet-based multidisciplinary online medical consultation system to help cope with pediatric medical needs during the COVID-19 outbreak: a cross-sectional study. *Transl Pediatr.* (2021) 10:560–8. 10.21037/tp-20-348 33850814 PMC8039788

[37] Olson CA McSwain SD Curfman AL Chuo J. The current pediatric telehealth landscape. *Pediatrics.* (2018) 141:e20172334. 10.1542/peds.2017-2334 29487164

[38] Nguyen M Waller M Pandya A Portnoy JA. Review of patient and provider satisfaction with telemedicine. *Curr Allergy Asthma Rep.* (2020) 20:72. 10.1007/s11882-020-00969-7 32959158 PMC7505720

